# Spinal cord diffusion tensor imaging predicts balance in degenerative cervical myelopathy

**DOI:** 10.3389/fnins.2026.1911141

**Published:** 2026-08-28

**Authors:** Viprav B. Raju, Roxanne Hauer, Mohamed Jalloh, Anjishnu Banerjee, Matthew Budde, Kevin M. Koch, Brian D. Schmit, Aditya Vedantam

**Affiliations:** 1 Department of Neurosurgery, Medical College of Wisconsin, Milwaukee, WI, United States; 2 Joint Department of Biomedical Engineering, Marquette University-Medical College of Wisconsin, Milwaukee, WI, United States; 3 Department of Biostatistics, Medical College of Wisconsin, Milwaukee, WI, United States; 4 Applied Sciences Institute, Hospital for Special Surgery, New York, NY, United States

**Keywords:** balance, biomarker, degenerative cervical myelopathy, diffusion tensor imaging, gait, postural stability, spinal cord, surgical outcomes

## Abstract

**Introduction:**

Degenerative cervical myelopathy (DCM) is the leading cause of spinal cord dysfunction in adults and a major contributor to balance impairment and gait instability. Surgical decompression halts disease progression, but predicting post-surgical functional recovery remains challenging. Conventional MRI poorly reflects spinal cord microstructural integrity, limiting its ability to quantify sensorimotor deficits or predict outcomes. Diffusion tensor imaging (DTI) provides quantitative measures of tract-specific microstructure and may serve as a more sensitive biomarker for functional impairment and recovery in DCM.

**Methods:**

In a prospective longitudinal cohort of 57 DCM patients undergoing surgical decompression, pre-operative spinal cord DTI was acquired at 3T. DTI metrics: fractional anisotropy (FA), mean diffusivity (MD), radial diffusivity (RD), axial diffusivity (AD), and filtered apparent diffusion coefficient (D_axial_) were extracted from dorsal, lateral, and ventral funiculi and gray matter at the C2 level. Functional outcomes included the Berg Balance Scale (BBS), Functional Gait Assessment (FGA), 10-Meter Walk Test (10MWT), and Eyes Closed Feet Together (ECFT) test, assessed pre-operatively and at 3 months post-surgery. General linear models examined baseline DTI–function associations; linear mixed-effects models assessed DTI prediction of post-operative improvement. Covariates were selected by pre-screening at *p* < 0.10.

**Results:**

Dorsal column FA (*p* = 0.034, *q* = 0.086) and RD (*p* = 0.025, *q* = 0.086) were associated with baseline BBS. Dorsal column MD (*p* = 0.010, *q* = 0.026) and RD (*p* = 0.009, *q* = 0.026) predicted post-operative BBS improvement. Lateral funiculi Daxial (*p* = 0.008, *q* = 0.038) predicted post-operative FGA improvement. Gray matter FA was associated with baseline FGA (*p* = 0.014, *q* = 0.070). Ventral funiculi metrics showed no significant associations. Forty-nine participants completed 3-month follow-up.

**Conclusion:**

Pre-operative C2-level DTI metrics demonstrate tract-specific associations with balance and gait function in DCM. Dorsal column microstructure was most strongly associated with static balance, while lateral funiculi and gray matter microstructure related more closely to dynamic balance and gait. These findings support spinal cord DTI as a quantitative imaging biomarker for functional prognostication and post-surgical outcome prediction in DCM.

## 1 Introduction

Degenerative cervical myelopathy (DCM) is the leading cause of spinal cord dysfunction in older adults worldwide (Badhiwala et al., 2020; Sial et al., 2024) and a major contributor to balance impairment, gait instability, and falls (Connelly et al., 2024). Surgical decompression is the standard of care to halt disease progression, but predicting the degree of post-surgical balance function remains challenging (Badhiwala et al., 2020; Connelly et al., 2024). Postural instability and gait dysfunction in DCM are multifactorial, arising from both sensory and motor pathway compromise (Boerger et al., 2024). The dorsal columns, which convey proprioceptive and tactile input, are critical for balance control, while the lateral funiculi carry descending corticospinal and vestibulospinal fibers important for dynamic gait and postural adjustment (Valošek et al., 2021). The ventral funiculi, by contrast, primarily contain motor pathways less directly involved in balance control (Chen et al., 2022). Autopsy (Dohle et al., 2023) and neuropathological studies (Chen et al., 2022) indicate that microstructural degeneration in DCM preferentially affects dorsal and lateral tracts, along with gray matter atrophy that disrupts sensorimotor integration. Additionally, a substantial proportion of DCM patients have balance-relevant non-DCM comorbidities that may independently affect balance and gait performance and this impacts post-surgical recovery of function. Predicting post-surgical balance and gait performance is necessary to improve pre-surgical decision-making, patient counseling and post-surgical rehabilitation planning. However, this remains an unmet clinical need and current clinical tools are unable to accurately prognosticate gait and balance function in DCM.

Conventional MRI (or clinical MRI) relies on T_2_- and T_1_-weighting imaging, which reveals the level and extent of cord compression as well as intramedullary changes such as T2-hyperintensities. However, conventional MRI does not quantify microstructural integrity of the spinal cord, limiting its ability to accurately predict tract-specific degeneration (Vedantam et al., 2014; Freund et al., 2019; Badhiwala et al., 2020). Diffusion tensor imaging (DTI) provides quantitative measures of spinal cord microstructure that consistently show alterations in the dorsal and lateral tracts of the cervical spinal cord in DCM patients, which correlate with clinical disability (Valošek et al., 2021; Al-Shawwa et al., 2024). DTI captures microstructural injury in regions of the spinal cord that appear normal on conventional MRI. Specifically, DTI metrics including fractional anisotropy (FA), mean diffusivity (MD), radial diffusivity (RD), and axial diffusivity (AD), are sensitive to axonal and myelin integrity and reflect likely axonal degeneration and demyelination (Vedantam et al., 2014; McLachlin et al., 2021). Prior studies in DCM have linked cervical spinal cord DTI to global disability measures such as the modified Japanese Orthopaedic Score (mJOA) (Gao et al., 2013; He et al., 2022; Grabher et al., 2016; d'Avanzo et al., 2020) (Ellingson et al., 2018; Martin et al., 2018). However, the mJOA is a coarse sensorimotor score that does not capture detailed balance, gait, or postural control deficits which are common in DCM and often the primary indication for surgery.

This study addresses two key questions: (1) Is spinal cord microstructural injury associated with baseline functional balance and gait performance in DCM? and (2) Can pre-surgical spinal cord DTI metrics predict early post-surgical improvements in these functions? We hypothesized that pre-surgical microstructural integrity in the dorsal columns and lateral funiculi would correlate with baseline balance and predict recovery at 3 months following decompression.

## 2 Methods

### 2.1 Study design

We conducted a prospective single-center observational study with pre-surgical DCM participants from March 2024 to September 2025. All participants provided informed consent before enrollment. The inclusion criteria were pre-surgical DCM patients (ages 18–90 years) with one or more clinical signs or symptoms of DCM, in addition to evidence of cervical spinal cord compression on MRI. Participants had to have at least one clinical sign of DCM, such as corticospinal motor deficits, hand atrophy, hyperreflexia, a positive Hoffman sign, upgoing plantar reflexes, lower limb spasticity, or gait ataxia; and at least one clinical symptom of DCM, including numb or clumsy hands, gait impairment, bilateral hand paresthesia, L'Hermitte's phenomenon, or weakness. We excluded patients with trauma, syringomyelia, cord hemorrhage, tumor, pregnancy, and other neurological or muscular diseases that could explain their symptoms, as well as those with a history of prior cervical spine surgery. Demographic data, co-morbidity data and clinical physical examination findings were recorded.

Relevant co-morbidities were coded as 1 if the participant had any balance-relevant non-DCM comorbidity or domain identified by clinical chart review, including: cerebrovascular or peripheral vascular disease; vestibular disorders, dizziness, syncope, POTS, or dysautonomia; peripheral neuropathy, distal sensory loss, neurogenic weakness,; visual impairment; diabetic peripheral neuropathy phenotype (diabetes or prediabetes co-occurring with neuropathy, distal sensory loss, diabetic ulcer, or toe amputation); lumbar stenosis, radiculopathy, sciatica, or prior lumbar surgery; and lower-extremity orthopedic disease or hip/knee osteoarthritis. Domains were not mutually exclusive; the binary covariate was 1 if any domain was present.

### 2.2 Functional measures

Four validated functional outcome measures and the mJOA questionnaire were assessed at baseline (pre-surgery) and 3 months post-surgery to quantify gait and balance performance. *Gait speed* was measured using the 10-Meter Walk Test (10MWT) (Watson, 2002), in which participants walked a 10-meter path at a comfortable pace, and the time taken to traverse the middle 6 meters was recorded to calculate gait speed (m/s). Balance performance was evaluated using the *Berg Balance Scale (BBS*) (Berg et al., 1992), a 14-item measure of static and dynamic balance scored on a 5-point scale (0–4), with higher scores indicating better balance, and the *Functional Gait Assessment (FGA)* (Kahn et al., 2020), a 10-item test assessing dynamic walking balance under various conditions such as head turns and obstacle negotiation, with a maximum possible score of 30. Postural stability was further quantified using the *Eyes Closed Feet Together (ECFT) task* (Romberg, 1853), during which participants stood with feet together and eyes closed for 60 s on a pressure-sensing platform (GaitRite Walkway, CIR Systems, Franklin NJ, USA); the total center of pressure (CoP) path length (in cm) was computed as an indicator of sway magnitude, with greater values reflecting poorer balance control. Demographic data, co-morbidity data and clinical physical examination findings were recorded. Disease severity was quantified using the mJOA scale (Benzel et al., 1991). The mJOA is a clinical symptom score that was included to contextualize DTI–function associations in this study.

### 2.3 MRI acquisition and analysis

MRI was performed on a 3T GE Premier prior to surgery, and all scans used a commercial 21-channel head and neck coil. The MRI protocol was based off the consensus spine protocol (Cohen-Adad et al., 2021) including a T_2_-weighted 3D fast spin-echo with TR = 2,500 ms, TE = 120 ms, field-of-view = 256 × 256 × 51 mm^3^, resolution = 0.5 × 0.5 × 0.8 mm^3^. The diffusion weighted imaging (DWI) protocol used a reduced field-of-view excitation (FOCUS) and echo planar imaging (EPI) readout with: TR = 4,000 ms, TE = 55 ms, NEX = 1, slice thickness = 5 mm, number of slices = 16, and in-plane resolution of 1.1 mm^2^. Diffusion weighting used 4 non-diffusion weighted images (b = 0 s/mm^2^) and 3 b-values (150, 1,000, 2,000 s/mm^2^) along 15, 30, and 30 directions, respectively. A separate DWI acquisition used a filtered DWI to suppress extracellular/extraspinal fluid with diffusion weighting perpendicular (left-right) to the cord (2,000 s/mm^2^) and lower diffusion weighting (500 s/mm^2^) along 20 orthogonal directions (Lee et al., 2021). The total acquisition time was 6 min 12 s for DWI scans.

**Figure 1:**
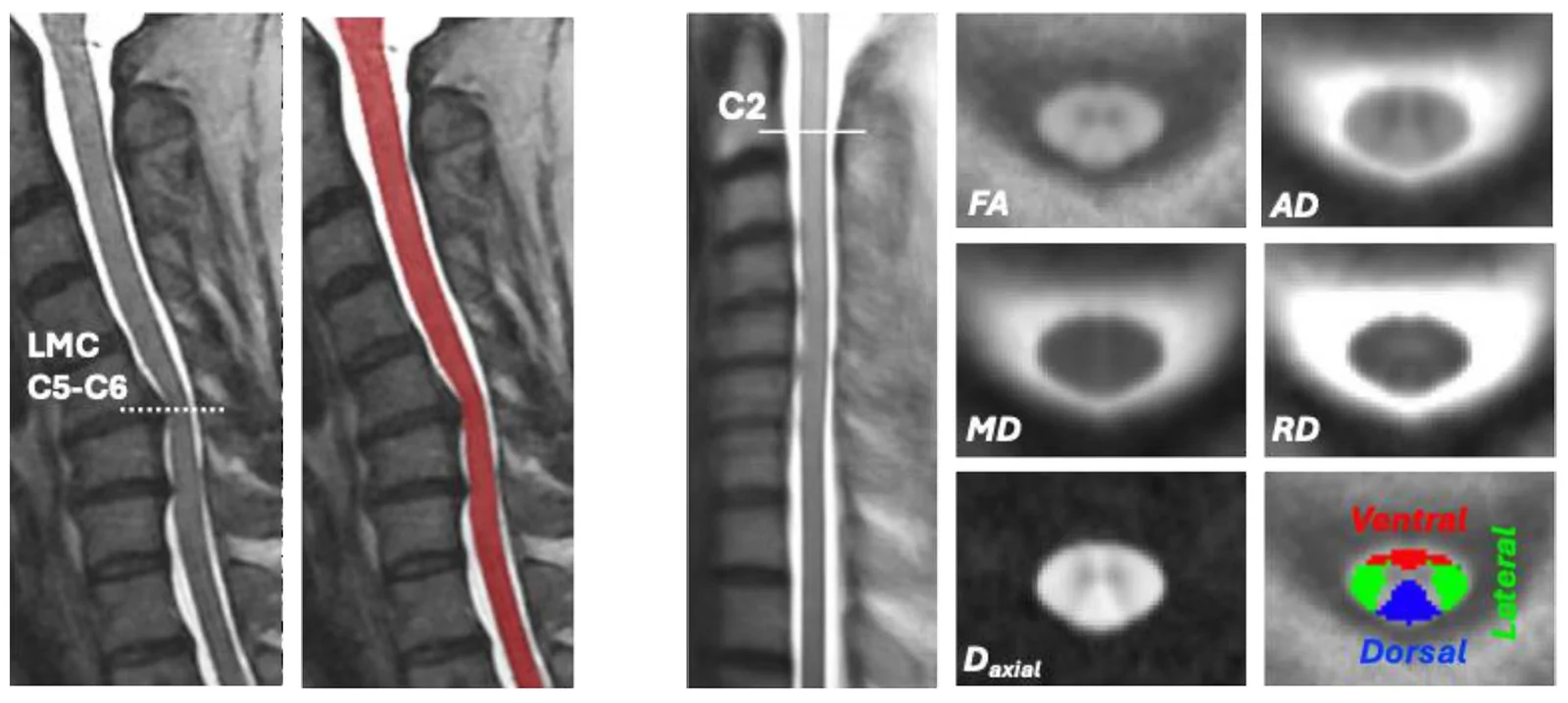
Spinal cord diffusion registration and regions of interest. A sagittal T_2_w image **(Left)** shows the cervical spinal cord of one subject, with automated spinal cord segmentation (red). The mean T_2_w image from all subjects shows the registration accuracy, with C2 indicated as the level for DTI metrics **(Right)**, including fractional anisotropy (FA), axial diffusivity (AD), mean diffusivity (MD), radial diffusivity (RD), and filtered ADC (D_axial_). The template-based anatomical regions of interest in ventral, dorsal, and lateral white matter are shown overlaid on the mean FA map. LMC- level of maximum compression.

Image analysis used off-line processing with pipelines developed in *nipype* in combination with the Spinal Cord Toolbox (v6.5) (De Leener et al., 2017). DWIs were denoised using an MPPCA algorithm and corrected for motion (Veraart et al., 2016). Maps of fractional anisotropy (FA), mean diffusivity (MD), axial diffusivity (AD), and radial diffusivity (RD) were estimated using a tensor model, and maps of filtered ADC (D_axial_) were estimated using a 2D tensor model, both using *dipy*. All images were aligned to a common template (PAM50) for quantification (Figure 1) (De Leener et al., 2018). The spinal cord was automatically segmented separately for the T_2_w image and the mean of all DW images (Bédard et al., 2025) (*sct_deepseg: contrast agnostic*). Further, the T_2_w image was segmented using an automated algorithm (*sct_deepseg: totalspineseg*) to automatically obtain landmarks at each cervical disc level (Warszawer et al., 2024). These disc references were used to initialize affine registration to the template. Cord segmentation (with manual review) masks were used for final non-linear warping to the template, constrained to the axial plane.

DWI metrics were averaged from regions of interest at the C2 level in template-space, including gray matter and dorsal, ventral, and lateral white matter (see Figure 1). DTI data quality was assessed by visual inspection of the derived fractional anisotropy (FA) maps for imaging artifacts and processing errors. No quantitative image quality metrics were used, however, our average coefficient of variation was 8% for all metrics ensuring adequate DTI quality. One axial diffusivity (D_axial_) value was excluded from the analysis because it was identified as an extreme outlier.

### 2.4 Statistical analysis

A univariate analysis was performed to determine the association between covariates (age, sex, symptom duration, T2-hyperintensity and comorbidity indicator) and each functional outcome. Covariates with *p* < 0.01 were included with the DTI metric in the multivariable models to predict baseline and post-surgical functional outcome. To examine the relationship between baseline DTI metrics and baseline functional performance, we performed ordinary least squares linear regression for each combination of DTI metric, anatomical tract, and functional outcome. To determine whether baseline DTI metrics predicted functional improvement following surgery, we used linear mixed effects models with participant as a random effect. Mixed effects models were fitted using restricted maximum likelihood estimation with the LBFGS (Limited-memory Broyden–Fletcher–Goldfarb–Shanno) (Nocedal, 1980) optimization method. To control for multiple comparisons, we applied the Benjamini-Hochberg (BH) false discovery rate (FDR) (Benjamini and Hochberg, 1995) correction separately for each tract × functional outcome combination across the five-diffusion metrics. FDR correction was applied independently for baseline regression models and mixed effects models. Multiplicity correction was performed by using the BH procedure, with an FDR control level of *q* = 10%. Statistical significance was defined as associations that met both the nominal significance threshold (*p* < 0.05) and the FDR criterion (*q* < 0.10).

All analyses were conducted using Python 3.10 (Python Software Foundation, Wilmington, DE, USA) with the following packages: *pandas* for data manipulation, *numpy* for numerical operations, and *statsmodels* for regression modeling (*OLS* and mixed effects models). Power was informed by prior work (Vedantam et al., 2014) demonstrating a moderate association between pre-operative fractional anisotropy and 3-month change in mJOA score (*r* = 0.42) in patients with cervical spondylotic myelopathy. Using this effect size as a conservative estimate for the MRI Metric × Time interaction in a two-timepoint linear mixed-effects model, and assuming approximately 49 participants with complete pre–post data and α = 0.05, the study has approximately 85–90% power to detect a moderate interaction effect (β*3* ≈ 0.4) between baseline MRI metrics and post-operative change in functional outcomes. Power is reduced for smaller effects, indicating the study is primarily powered to detect clinically meaningful moderate associations.

## 3 Results

**Figure 2:**
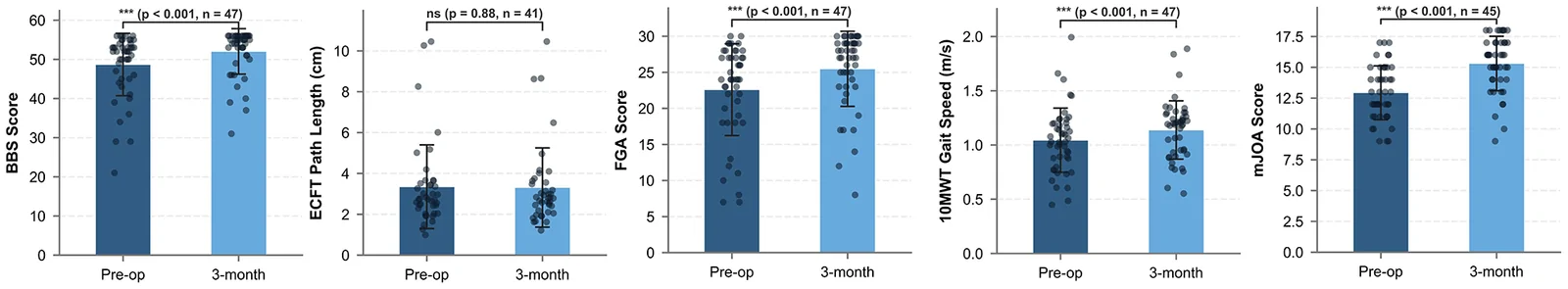
Pre-operative and 3-month post-operative functional outcomes. Bar graphs show group mean ± SD for each functional measure at baseline (pre-operative, dark blue) and 3 months post-surgery (light blue). Individual data points are overlaid. Significance brackets indicate paired *t*-test results with the asterisk notation (*p < 0.05, **p < 0.01, ***p < 0.001) and sample size. BBS, berg balance scale; FGA, functional gait assessment; 10MWT, 10-meter walk test gait speed (m/s); ECFT, eyes closed feet together COP path length (cm); mJOA, modified Japanese orthopaedic association scale.

**Figure 3:**
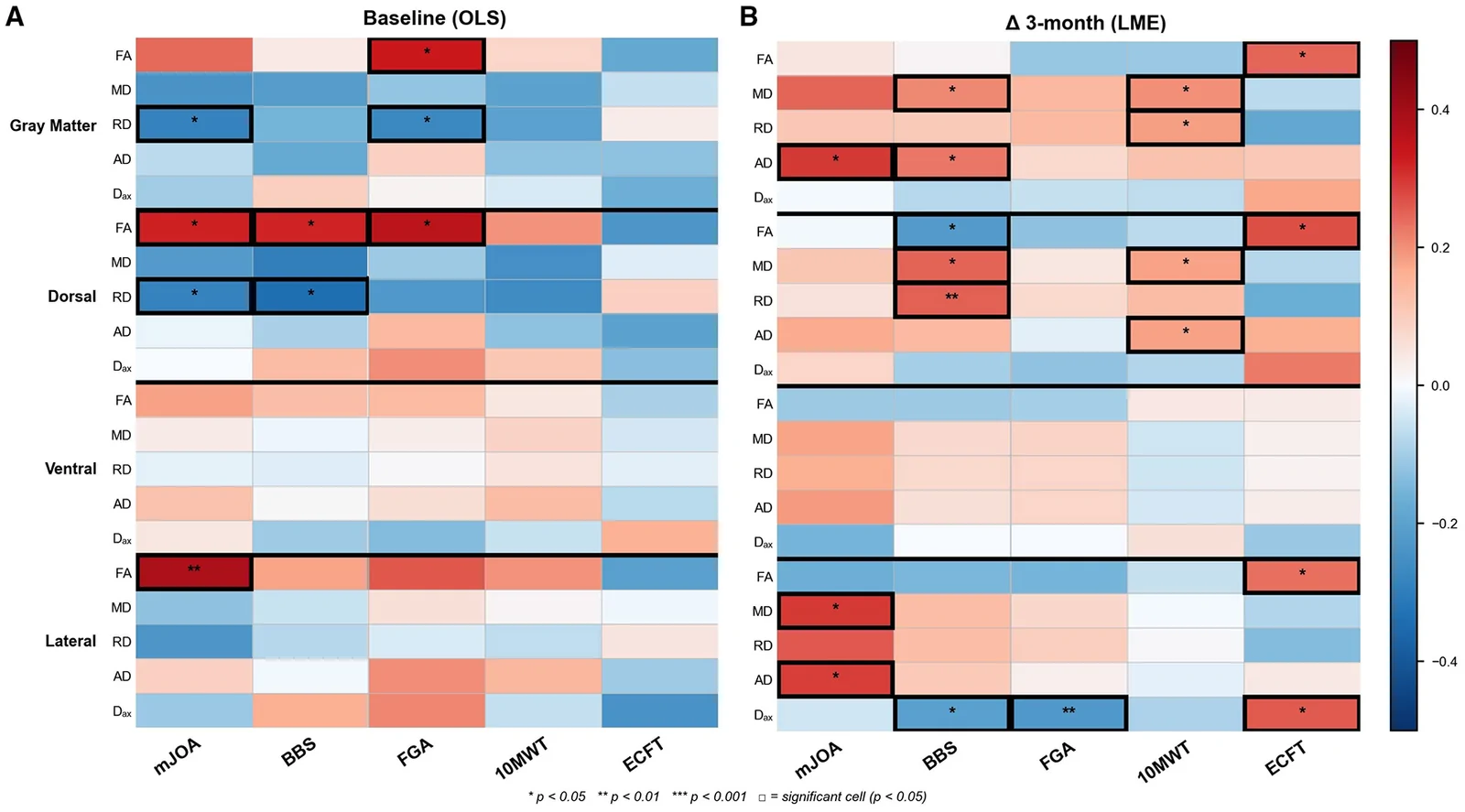
Heat maps of standardized β coefficients (C2 level, binary balance comorbidity covariate). Each heat map shows 20 rows (four anatomical regions × five diffusion metrics: FA, MD, RD, AD, D_axial_) against six columns (functional outcomes: mJOA, BBS, FGA, 10MWT, ECFT). Red tones indicate positive β coefficients (higher MRI metric associated with better outcome); blue tones indicate negative β coefficients. Thick black horizontal lines separate anatomical regions (Gray Matter, Dorsal, Ventral, Lateral). Cells with statistically significant associations (*p* < 0.05) are denoted by asterisks (*p < 0.05, **p < 0.01, ***p < 0.001) and outlined with a black border. **(A)** Baseline OLS models. **(B)** Δ 3-month LME models.

**Figure 4:**
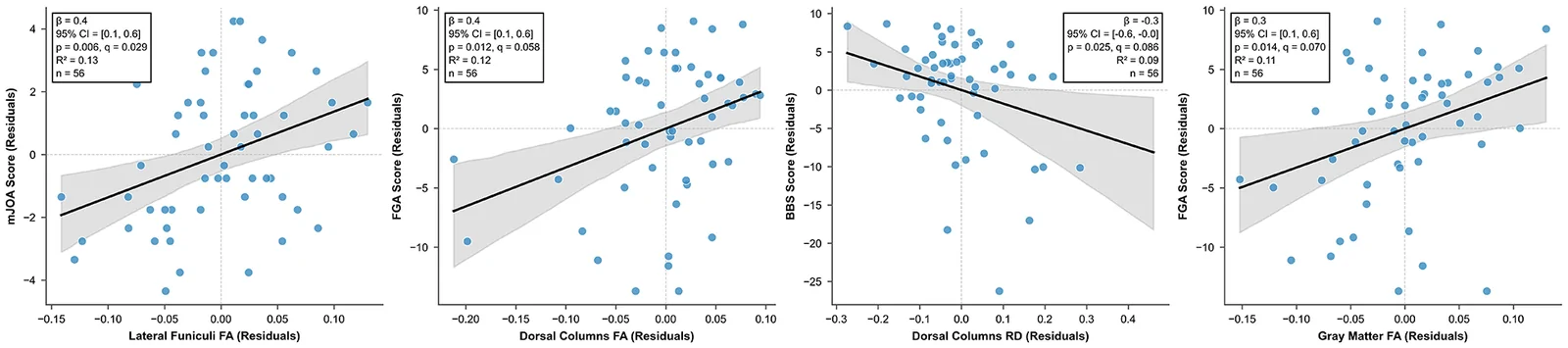
Associations between cervical spinal cord microstructure and functional scores. Adjusted partial regression plots demonstrate relationships between diffusion MRI metrics and functional performance. Each panel illustrates a significant association that survived false discovery rate (FDR) correction at *q* < 0.1. The *x*-axis represents residualized MRI metrics, while the *y*-axis represents residualized functional scores. Shaded regions denote 95% bootstrap confidence intervals for the regression line. Each panel is labeled with the standardized β coefficient, 95% CI, *p*-value, FDR-corrected *q*-value, *R*^2^, and sample size. Only the four associations with the largest standardized effect sizes are shown.

The study included 57 DCM participants with baseline functional assessments and MRI data (Table 1 and Figure 2). Forty-nine participants had gait and balance function assessments at 3 months post-surgery (8 had missing follow-up data, including participants lost to attrition, withdrawn, or deceased), yielding 98 observations for the longitudinal mixed-effects model. Sample sizes varied slightly due to missing MRI metrics or functional outcome data. For ECFT, eight observations were missing (5 due to technical issues, 2 due to inability to perform the test, and 1 due to incomplete assessment), resulting in ECFT data from 49 participants at baseline and 41 participants post-operatively. Comparison of baseline clinical and imaging variables between 49 patients with follow-up and 8 patients without follow-up showed a lower baseline Berg Balance Score compared to the group with follow-up data. However, there were no systematic differences in the other clinical and imaging variables, indicating that attrition is unlikely to have influenced Type I error (see Supplementary Table 1). Covariate pre-screening identified age and comorbidity covariate as the most broadly influential covariates across functional outcomes. In our cohort, 23 of 57 participants (40%) had at least one relevant comorbidity including cerebrovascular or peripheral vascular disease, vestibular dysfunction, peripheral neuropathy, visual impairment, diabetic peripheral neuropathy, lumbar stenosis, or lower-extremity orthopedic conditions. Age met the inclusion threshold (*p* < 0.10) for BBS, FGA, and 10MWT models, while comorbidity covariate was retained for BBS, 10MWT, and ECFT models. Sex and symptom duration were included only in the 10MWT model. No covariates met the pre-screening threshold for mJOA. The results are summarized in Table 2 and Figures 3, 4.

**Table 1:** Participant demographics and baseline clinical characteristics.

Variable	Mean ±SD/*n* (%)
Total	*n* = 57
Males	33 (57.9%)
Females	24 (42.1%)
Age	59.2 ± 12.1 yrs
Balance comorbidity	23 (40%)
Baseline functional measure	
BBS (*n* = 56)	48.48 ± 7.8
ECFT COP path length (*n* = 52)	3.85 ± 2.5 cm
FGA (*n* = 56)	22.36 ± 6.3
10MWT gait speed (*n* = 56)	1.05 ± 0.3 m/s
Baseline mJOA	
mJOA (*n* = 56)	13 ± 2.2
mJOA severity	
- Mild	16 (28.6%)
- Moderate	24 (42.9%)
- Severe	16 (28.6%)
Duration of follow-up (*n* = 49)	3.39 ± 0.36 months

**Table 2:** Associations between spinal cord microstructural metrics and functional scores.

Column	Functional measure	Timepoint	FA	MD	RD	AD	D_axial_
Dorsal	mJOA	Baseline	0.323 [0.05, 0.60]	−0.215 [−0.49, 0.06]	−0.285 [−0.56, −0.01]	−0.017 [−0.29, 0.26]	0.001 [−0.29, 0.29]
Dorsal	mJOA	Δ 3 months	−0.004 [−0.28, 0.27]	0.115 [−0.16, 0.39]	0.056 [−0.22, 0.33]	0.170 [−0.10, 0.44]	0.082 [−0.20, 0.36]
Dorsal	BBS	Baseline	**0.321 [0.02, 0.62]**	−0.295 [−0.59, 0.00]	**−0.339 [−0.63**, **−0.04]**	−0.090 [−0.39, 0.21]	0.136 [−0.14, 0.41]
Dorsal	BBS	Δ 3 months	**−0.215 [−0.41**, **−0.02]**	**0.249 [0.06, 0.44]**	**0.253 [0.06, 0.44]**	0.143 [−0.06, 0.34]	−0.097 [−0.30, 0.11]
Dorsal	FGA	Baseline	**0.358 [0.08, 0.63]**	−0.106 [−0.37, 0.16]	−0.224 [−0.49, 0.04]	0.142 [−0.13, 0.42]	0.202 [−0.07, 0.48]
Dorsal	FGA	Δ 3 months	−0.121 [−0.29, 0.05]	0.045 [−0.13, 0.22]	0.074 [−0.10, 0.25]	−0.024 [−0.20, 0.15]	−0.121 [−0.30, 0.05]
Dorsal	10MWT	Baseline	0.196 [−0.10, 0.49]	−0.250 [−0.54, 0.04]	−0.260 [−0.55, 0.03]	−0.124 [−0.41, 0.16]	0.112 [−0.16, 0.39]
Dorsal	10MWT	Δ 3 months	−0.072 [−0.25, 0.11]	0.179 [0.01, 0.35]	0.136 [−0.04, 0.31]	0.181 [0.01, 0.35]	−0.082 [−0.26, 0.10]
Dorsal	ECFT	Baseline	−0.231 [−0.52, 0.06]	−0.028 [−0.35, 0.30]	0.092 [−0.22, 0.41]	−0.198 [−0.48, 0.09]	−0.130 [−0.41, 0.15]
Dorsal	ECFT	Δ 3 months	**0.272 [0.06, 0.49]**	−0.077 [−0.31, 0.15]	−0.167 [−0.39, 0.06]	0.161 [−0.06, 0.39]	0.223 [−0.00, 0.45]
Lateral	mJOA	Baseline	**0.381 [0.11, 0.65]**	−0.121 [−0.40, 0.16]	−0.232 [−0.51, 0.05]	0.091 [−0.18, 0.36]	−0.108 [−0.39, 0.17]
Lateral	mJOA	Δ 3 months	−0.165 [−0.44, 0.11]	**0.295 [0.03, 0.56]**	0.260 [−0.01, 0.53]	**0.290 [0.03, 0.55]**	−0.045 [−0.33, 0.24]
Lateral	BBS	Baseline	0.177 [−0.11, 0.46]	−0.054 [−0.32, 0.22]	−0.077 [−0.36, 0.20]	−0.007 [−0.27, 0.25]	0.162 [−0.11, 0.43]
Lateral	BBS	Δ 3 months	−0.148 [−0.35, 0.05]	0.135 [−0.07, 0.34]	0.133 [−0.07, 0.33]	0.107 [−0.10, 0.31]	−0.204 [−0.40, −0.01]
Lateral	FGA	Baseline	0.262 [−0.01, 0.53]	0.060 [−0.21, 0.33]	−0.036 [−0.31, 0.24]	0.202 [−0.05, 0.46]	0.210 [−0.06, 0.48]
Lateral	FGA	Δ 3 months	−0.151 [−0.32, 0.02]	0.078 [−0.10, 0.25]	0.097 [−0.08, 0.27]	0.026 [−0.15, 0.20]	**−0.225 [−0.39**, **−0.06]**
Lateral	10MWT	Baseline	0.196 [−0.08, 0.47]	0.016 [−0.27, 0.30]	−0.065 [−0.35, 0.22]	0.145 [−0.13, 0.42]	−0.059 [−0.33, 0.21]
Lateral	10MWT	Δ 3 months	−0.058 [−0.24, 0.12]	−0.003 [−0.18, 0.18]	0.008 [−0.17, 0.19]	−0.022 [−0.20, 0.16]	−0.088 [−0.27, 0.09]
Lateral	ECFT	Baseline	−0.203 [−0.48, 0.07]	−0.009 [−0.29, 0.27]	0.050 [−0.23, 0.33]	−0.105 [−0.37, 0.16]	−0.242 [−0.51, 0.03]
Lateral	ECFT	Δ 3 months	0.236 [0.02, 0.45]	−0.081 [−0.31, 0.15]	−0.135 [−0.36, 0.09]	0.040 [−0.19, 0.27]	**0.259 [0.04, 0.48]**
Ventral	mJOA	Baseline	0.182 [−0.09, 0.45]	0.036 [−0.24, 0.31]	−0.021 [−0.30, 0.25]	0.124 [−0.15, 0.40]	0.044 [−0.23, 0.32]
Ventral	mJOA	Δ 3 months	−0.106 [−0.38, 0.17]	0.177 [−0.10, 0.45]	0.163 [−0.11, 0.44]	0.189 [−0.08, 0.46]	−0.151 [−0.43, 0.13]
Ventral	BBS	Baseline	0.132 [−0.14, 0.40]	−0.014 [−0.27, 0.25]	−0.029 [−0.29, 0.23]	0.010 [−0.25, 0.27]	−0.105 [−0.36, 0.16]
Ventral	BBS	Δ 3 months	−0.106 [−0.31, 0.10]	0.071 [−0.13, 0.27]	0.076 [−0.13, 0.28]	0.060 [−0.14, 0.26]	0.001 [−0.21, 0.21]
Ventral	FGA	Baseline	0.139 [−0.13, 0.41]	0.030 [−0.23, 0.29]	0.006 [−0.26, 0.27]	0.064 [−0.19, 0.32]	−0.137 [−0.40, 0.12]
Ventral	FGA	Δ 3 months	−0.096 [−0.27, 0.08]	0.084 [−0.09, 0.26]	0.082 [−0.09, 0.26]	0.083 [−0.09, 0.26]	−0.001 [−0.18, 0.18]
Ventral	10MWT	Baseline	0.044 [−0.22, 0.31]	0.088 [−0.17, 0.35]	0.054 [−0.21, 0.31]	0.136 [−0.12, 0.39]	−0.055 [−0.31, 0.20]
Ventral	10MWT	Δ 3 months	0.042 [−0.14, 0.22]	−0.046 [−0.22, 0.13]	−0.047 [−0.23, 0.13]	−0.043 [−0.22, 0.14]	0.057 [−0.12, 0.24]
Ventral	ECFT	Baseline	−0.091 [−0.36, 0.18]	−0.044 [−0.31, 0.22]	−0.024 [−0.29, 0.24]	−0.073 [−0.34, 0.20]	0.157 [−0.11, 0.42]
Ventral	ECFT	Δ 3 months	0.036 [−0.19, 0.27]	0.025 [−0.21, 0.26]	0.020 [−0.21, 0.25]	0.032 [−0.20, 0.26]	−0.110 [−0.34, 0.12]
Gray matter	mJOA	Baseline	0.241 [−0.03, 0.51]	−0.240 [−0.51, 0.03]	−0.285 [−0.55, −0.02]	−0.069 [−0.34, 0.20]	−0.100 [−0.38, 0.18]
Gray matter	mJOA	Δ 3 months	0.047 [−0.23, 0.32]	0.248 [−0.02, 0.52]	0.111 [−0.16, 0.39]	0.296 [0.03, 0.56]	−0.002 [−0.28, 0.28]
Gray matter	BBS	Baseline	0.038 [−0.24, 0.32]	−0.213 [−0.48, 0.06]	−0.153 [−0.44, 0.13]	−0.176 [−0.43, 0.08]	0.097 [−0.17, 0.37]
Gray matter	BBS	Δ 3 months	0.017 [−0.19, 0.22]	**0.209 [0.01, 0.40]**	0.104 [−0.10, 0.31]	**0.227 [0.03, 0.42]**	−0.076 [−0.28, 0.13]
Gray matter	FGA	Baseline	**0.335 [0.07, 0.60]**	−0.118 [−0.38, 0.14]	−0.272 [−0.54, −0.01]	0.092 [−0.17, 0.35]	0.018 [−0.26, 0.29]
Gray matter	FGA	Δ 3 months	−0.114 [−0.29, 0.06]	0.143 [−0.03, 0.31]	0.142 [−0.03, 0.31]	0.073 [−0.10, 0.25]	−0.057 [−0.23, 0.12]
Gray matter	10MWT	Baseline	0.081 [−0.21, 0.37]	−0.198 [−0.46, 0.06]	−0.199 [−0.48, 0.08]	−0.123 [−0.39, 0.14]	−0.038 [−0.30, 0.22]
Gray matter	10MWT	Δ 3 months	−0.109 [−0.28, 0.07]	**0.201 [0.03, 0.37]**	**0.183 [0.01, 0.35]**	0.123 [−0.05, 0.30]	−0.067 [−0.25, 0.11]
Gray matter	ECFT	Baseline	−0.180 [−0.45, 0.09]	−0.059 [−0.34, 0.22]	0.031 [−0.25, 0.32]	−0.124 [−0.39, 0.14]	−0.166 [−0.43, 0.10]
Gray matter	ECFT	Δ 3 months	0.247 [0.03, 0.46]	−0.070 [−0.30, 0.16]	−0.187 [−0.41, 0.04]	0.109 [−0.12, 0.34]	0.170 [−0.06, 0.40]

Standardized beta regression coefficients [95% CIs] are shown for baseline and 3–month changes (Δ) across four functional measures: BBS, ECFT path length, FGA, and gait speed. Baseline associations were assessed using general linear models (GLM), while longitudinal changes were assessed using linear mixed-effects models (LME). Values highlighted in bold indicate significance at false discovery rate (FDR) *q* < 0.1.

### 3.1 Dorsal columns

At baseline, dorsal column microstructural integrity was significantly associated with balance performance measured by the BBS. Specifically, FA (*p* = 0.034, *q* = 0.086) and RD (*p* = 0.025, *q* = 0.086) were associated with baseline BBS. Baseline FGA was significantly associated with FA (*p* = 0.012, *q* = 0.058). Longitudinally, improvement in BBS over 3 months was significantly associated with dorsal column FA (*p* = 0.030, *q* = 0.051), MD (*p* = 0.010, *q* = 0.026), and RD (*p* = 0.009, *q* = 0.026). Improvement in ECFT was significantly associated with dorsal column FA (*p* = 0.013, *q* = 0.065). No significant longitudinal associations were observed for FGA, mJOA, or 10MWT gait speed.

### 3.2 Lateral funiculi

For the lateral funiculi, baseline mJOA was significantly associated with FA (*p* = 0.006, *q* = 0.029). Longitudinally, improvement in FGA over 3 months was strongly associated with Daxial (*p* = 0.008, *q* = 0.038). Improvement in mJOA was associated with MD (*p* = 0.028, *q* = 0.079) and AD (*p* = 0.031, *q* = 0.079). Improvement in ECFT was significantly associated with FA (*p* = 0.035, *q* = 0.087) and D_axial_ (*p* = 0.021, *q* = 0.087). No significant longitudinal associations were observed for gait speed.

### 3.3 Ventral funiculi

No significant associations were observed between ventral funiculi microstructure and any functional outcomes.

### 3.4 Gray Matter

Gray matter microstructure demonstrated significant associations with baseline function and longitudinal recovery. Specifically, baseline FGA was significantly associated with FA (*p* = 0.014, *q* = 0.070). Longitudinally, improvement in BBS over 3 months was significantly associated with MD (*p* = 0.036, *q* = 0.090) and AD (*p* = 0.021, *q* = 0.090). Improvement in 10MWT gait speed was associated with MD (*p* = 0.020, *q* = 0.089) and RD (*p* = 0.036, *q* = 0.089). No significant baseline associations with BBS, gait speed or mJOA survived FDR correction at *q* < 0.10 in the model controlling for balance comorbidity.

## 4 Discussion

This study demonstrates the association between cervical spinal cord DTI metrics (measured rostral to the level of cord compression) and balance/gait function in DCM. In a cohort of 57 participants, all models controlled for balance-relevant non-DCM comorbidities, addressing a key confound in prior DTI–function studies. Microstructural integrity of the dorsal columns and gray matter emerged as the strongest predictors of balance and gait, with additional contributions from lateral funiculi. In contrast, ventral funiculi showed no significant associations with functional measures. Baseline tract integrity predicted post-surgical improvements, supporting the utility of spinal cord diffusion MRI as a biomarker for recovery of balance function after surgery for DCM.

The most consistent associations were observed in the dorsal columns and lateral funiculi, with minimal involvement of ventral tracts. The dorsal columns, responsible for proprioceptive feedback, were significantly associated with balance (BBS) performance. Reduced integrity in these sensory pathways was linked to poorer postural control and impaired recovery. The lateral funiculi, containing corticospinal tracts, were associated with FGA improvements, supporting their role in dynamic balance and gait adaptability. These findings parallel autopsy (Dohle et al., 2023) and neuroanatomical studies (Valošek et al., 2021; Chen et al., 2022), which show that DCM predominantly affects the dorsolateral white matter cranial to the compression, consistent with both sensorimotor functional impairments that affect gait and balance function. In contrast, ventral funiculi did not show significant associations with gait or balance outcomes. Ventral and lateral tracts may sustain damage *below* the compression site, where descending fibers are more susceptible to injury, but such changes would not be detectable rostral to the level of cord compression. Therefore, the weaker associations in ventral tracts could reflect variations in neurodegeneration in relation to the level of cord compression. Tract-specific microstructure, remote from the level of maximum spinal cord compression, could serve as a quantitative biomarker for recovery in key functional domains such as BBS and FGA. This study is the first to demonstrate a domain-specific link between spinal cord diffusion microstructure and functional recovery in balance and gait among patients with DCM.

DTI measures of the dorsal columns and gray matter predicted post-surgical improvements in both balance and gait, highlighting the prognostic potential of diffusion MRI for pre-surgical counseling and individualized outcome prediction. Traditional MRI findings, such as T2 hyperintensity, have shown limited prognostic value for balance function in DCM (Arvin et al., 2013). Arvin et al. (2013) reported that low T1 signal is associated with poor BBS scores (*P* < 0.001) at baseline, and pre-operative volume of the T2 signal hyperintensity demonstrated significant associations with BBS scores at follow-up (*P* < 0.001). Gray matter microstructure was linked to both gait speed and balance function in DCM. Individuals with lower gray matter MD, reflecting more preserved gray matter organization and cellular integrity (Akter et al., 2020; Nanda et al., 2022), demonstrated superior baseline function and greater recovery over time. These results align with findings from multiple sclerosis (Klaver et al., 2013) and traumatic spinal cord injury studies (David et al., 2019), where gray matter damage predicts motor impairment and recovery potential. In DTI, higher MD or RD indicates increased extracellular space, reflecting processes such as neuronal glial loss or edema, whereas lower diffusion values correspond to intact tissue organization and membrane integrity (Vedantam et al., 2014; Kaushal et al., 2019). This interpretation aligns with reports that gray matter damage, as characterized by elevated MD, predicts deficits in sensorimotor recovery in degenerative cervical myelopathy (Grabher et al., 2017; Seif et al., 2020). In DCM, gray matter degeneration above the level of maximum cord compression likely arises from a combination of chronic ischemia, interneuron loss, and reactive gliosis, due to retrograde axonal degeneration extending from compressed white matter tracts (Vedantam et al., 2014; Liu et al., 2020; Freund et al., 2024). Prior studies (Raghupathi, 2004; Algarni et al., 2024; David et al., 2025) have demonstrated that diffusion abnormalities at remote levels reflect secondary degeneration and correlate with clinical outcomes. Studies measuring DTI *cranial* to the level of compression have shown that diffusion imaging is sensitive to spinal cord injury in regions remote from the lesion (Jirjis et al., 2013; Motovylyak et al., 2019). Diffusion measures can detect microstructural injury that may not be visible on conventional imaging (Vedantam et al., 2014; Freund et al., 2019; Badhiwala et al., 2020). Collectively, these findings indicate that retrograde DTI changes in gray matter reflect microstructural injury contributing to sensorimotor dysfunction and impaired balance control.

This study has several limitations including the modest sample size and loss of follow-up to attrition. Sensitivity analyses comparing participants with and without three-month follow-up data found a lower baseline BBS in those lost to follow-up but no systematic differences in other clinical or imaging variables, suggesting attrition is unlikely to have biased the primary findings. The inclusion of balance comorbidity as a covariate strengthens causal inference, though the binary coding may not capture the full spectrum of comorbidity severity. The study is limited by lack of longer-term follow-up, however, 3 months may be sufficient to capture the early post-operative improvements in myelopathic symptoms (Khan et al., 2020). While imaging at C2 minimizes artifacts from cord compression and allows for accurate tract-specific DTI measurements, it may not reflect local tract disruption at the level of maximum compression. Prior DTI studies in DCM populations have used a similar approach highlighting the extent of retrograde neuronal injury in DCM (Vedantam et al., 2014; Freund et al., 2019; Badhiwala et al., 2020). Larger cohorts and longer follow-up durations will further clarify the predictive utility of these metrics for long-term recovery in DCM. To account for multiple testing, we applied Benjamini-Hochberg false discovery rate correction across the pre-specified family of tests and considered associations with *q* < 0.10 to be FDR-significant. We selected *q* < 0.10 because this was an exploratory discovery analysis in degenerative cervical myelopathy, a condition in which imaging and biomarker studies often involve correlated candidate features and modest sample sizes. The threshold was chosen to preserve sensitivity for potentially meaningful candidate signals while still controlling the expected proportion of false discoveries among findings declared significant. However, FDR control does not eliminate false-positive findings, and a *q* < 0.10 threshold implies that some reported discoveries may be false positives. Accordingly, these findings should be interpreted as candidate associations that require confirmation in independent cervical myelopathy cohorts, with attention to effect size, directionality, biological plausibility, and reproducibility.

An additional limitation is that the primary analyses focused on diffusion-weighted imaging (DWI)-derived metrics. Although complementary MRI biomarkers, including spinal cord morphology obtained from high-resolution 3D T2-weighted imaging (e.g., cord diameter and compression ratio) and magnetization transfer ratio (MTR), may provide additional information regarding structural injury and tissue integrity, these measures were not integrated into the present analysis. Future studies should investigate whether combining DTI metrics with morphological and MTR measures improves the characterization of disease severity and functional impairment. Advanced techniques such as spinal cord functional MRI (fMRI) may also provide complementary insights into functional reorganization within the spinal cord after surgical decompression.

## 5 Conclusion

In this prospective study, tract-specific pre-surgical DTI metrics above the level of maximal cord compression were associated with baseline and post-surgical gait/balance function in patients with DCM. Microstructural integrity of the dorsal columns was most strongly associated with static balance, whereas lateral funiculi and gray matter microstructure were more closely related to dynamic balance and gait performance. Cervical spinal cord DTI may serve as a promising biomarker for domain-specific functional outcomes in DCM.

## Data Availability

The datasets presented in this article are not readily available because summary results and data supporting the findings of this study are available from the corresponding author upon reasonable request, subject to ethical and institutional approvals. Requests to access the datasets should be directed to Aditya Vedantam, avedantam@mcw.edu.
