# Supplementary material for: Spinal cord diffusion tensor imaging predicts balance in degenerative cervical myelopathy
Source: Front Neurosci. 2026 Aug 28;20:1911141. doi: 10.3389/fnins.2026.1911141 (PMC13568261; doi:10.3389/fnins.2026.1911141)
Supplement: Supplementary file 1 [file Data_Sheet_1.docx]

Supplementary Material

# 1 Supplementary Table 1.

**Table 1. Sensitivity analysis: comparison of baseline characteristics between participants with and without three-month follow-up**

| Variable | With FU (n=49) Mean ± SD | Without FU (n=8) Mean ± SD | p-value | Test |
| --- | --- | --- | --- | --- |
| Age (years) | 59.24 ± 11.43 | 58.75 ± 16.80 | 0.938 | Welch's t |
| Sex (male=1) | 0.59 ± 0.50 | 0.50 ± 0.53 | 0.638 | Mann-Whitney U |
| Symptom duration (months) | 43.98 ± 52.90 | 28.25 ± 23.80 | 0.181 | Welch's t |
| mJOA baseline | 12.96 ± 2.23 | 13.25 ± 2.38 | 0.753 | Welch's t |
| BBS baseline | 48.77 ± 8.00 | 46.75 ± 6.11 | 0.426 | Welch's t |
| Balance comorbidity (binary) | 0.37 ± 0.49 | 0.62 ± 0.52 | 0.177 | Mann-Whitney U |
| C2 FA Dorsal | 0.63 ± 0.07 | 0.67 ± 0.04 | 0.031 | Welch's t |
| C2 FA Gray matter | 0.58 ± 0.07 | 0.56 ± 0.06 | 0.395 | Welch's t |
| C2 FA Lateral | 0.65 ± 0.06 | 0.67 ± 0.06 | 0.354 | Welch's t |

## Note: Continuous variables compared using Welch's independent-samples t-test; binary and ordinal variables compared using the Mann–Whitney U test. Data presented as mean ± SD. BBS, Berg Balance Scale; mJOA, modified Japanese Orthopaedic Association scale; FA, fractional anisotropy.

# 2 Supplementary Table 2.

**Table 2. C2-level Spinal Cord DTI Metrics — Baseline Summary (N = 57)**
Note: Values presented as Mean ± SD [Range: Min–Max]. FA = fractional anisotropy (dimensionless); MD, RD, AD = mean/radial/axial diffusivity (×10⁻³ mm²/s); Daxial = filtered apparent diffusion coefficient (dimensionless). n = 56 for D_axial_ (1 participant with outlier); n = 57 for all other metrics.

| Metric | Unit | Region | n | Mean | SD | Min | Max |
| --- | --- | --- | --- | --- | --- | --- | --- |
| FA | dimensionless | Whole cord | 57 | 0.6251 | 0.0555 | 0.4855 | 0.7276 |
|  |  | Dorsal columns | 57 | 0.6345 | 0.0685 | 0.3845 | 0.7565 |
|  |  | Lateral funiculi | 57 | 0.6505 | 0.0624 | 0.4886 | 0.7622 |
|  |  | Ventral funiculi | 57 | 0.5968 | 0.0863 | 0.3333 | 0.7465 |
|  |  | Gray matter | 57 | 0.5806 | 0.0636 | 0.4396 | 0.7373 |
| MD | ×10⁻³ mm²/s | Whole cord | 57 | 1.0544 | 0.1145 | 0.9043 | 1.5262 |
|  |  | Dorsal columns | 57 | 1.1045 | 0.1327 | 0.9103 | 1.5943 |
|  |  | Lateral funiculi | 57 | 1.0669 | 0.1419 | 0.8835 | 1.6283 |
|  |  | Ventral funiculi | 57 | 1.1088 | 0.3268 | 0.6590 | 2.4240 |
|  |  | Gray matter | 57 | 0.9181 | 0.0874 | 0.8115 | 1.2630 |
| RD | ×10⁻³ mm²/s | Whole cord | 57 | 0.6282 | 0.1214 | 0.4338 | 1.1007 |
|  |  | Dorsal columns | 57 | 0.6494 | 0.1479 | 0.4449 | 1.3116 |
|  |  | Lateral funiculi | 57 | 0.6155 | 0.1474 | 0.3929 | 1.1841 |
|  |  | Ventral funiculi | 57 | 0.6972 | 0.3033 | 0.4002 | 1.9856 |
|  |  | Gray matter | 57 | 0.5737 | 0.0884 | 0.4141 | 0.8913 |
| AD | ×10⁻³ mm²/s | Whole cord | 57 | 1.9070 | 0.1446 | 1.6787 | 2.3773 |
|  |  | Dorsal columns | 57 | 2.0148 | 0.1705 | 1.5522 | 2.5868 |
|  |  | Lateral funiculi | 57 | 1.9703 | 0.1647 | 1.6886 | 2.5167 |
|  |  | Ventral funiculi | 57 | 1.9319 | 0.3910 | 1.1725 | 3.3007 |
|  |  | Gray matter | 57 | 1.6068 | 0.1540 | 1.3548 | 2.1535 |
| D_axial_ | dimensionless | Whole cord | 56 | 0.9698 | 0.0101 | 0.9448 | 0.9953 |
|  |  | Dorsal columns | 56 | 0.9619 | 0.0205 | 0.9034 | 0.9963 |
|  |  | Lateral funiculi | 56 | 0.9632 | 0.0108 | 0.9438 | 0.9916 |
|  |  | Ventral funiculi | 56 | 0.9573 | 0.0339 | 0.8412 | 1.0394 |
|  |  | Gray matter | 56 | 1.0037 | 0.0194 | 0.9945 | 1.1427 |

# 3 Supplementary Figure 1

**Supplementary Figure 1. CONSORT Flow Diagram**
